# Perceptions of Nigerian Women about Human Papilloma Virus, Cervical Cancer, and HPV Vaccine

**DOI:** 10.1155/2015/285702

**Published:** 2015-10-13

**Authors:** Olusola Anuoluwapo Akanbi, Abiodun Iyanda, Folakemi Osundare, Oluyinka Oladele Opaleye

**Affiliations:** Department of Medical Microbiology and Parasitology, College of Health Sciences, Ladoke Akintola University of Technology, PMB 4400, Osogbo, Nigeria

## Abstract

*Background*. Cervical cancer caused by human papilloma virus (HPV) though preventable has claimed the lives of many women worldwide. This study was embarked upon to evaluate the general knowledge and perceptions of Nigerian women on HPV, cervical cancer, and HPV vaccine.* Methods*. Structured questionnaires were administered to a cross section of 737 women randomly selected from the general population in two southwestern States of Nigeria. Statistical analysis was done using SPSS computer software version 16. A *P* value >0.05 was considered statistically significant.* Results*. One hundred and seventy-six (23.9%) of the respondents had knowledge of HPV; 474 (64.3%) are aware of cervical cancer but only 136 (18.5%) know that HPV causes cervical cancer. 200 (27.1%) are aware that there is an HPV vaccine while 300 (40.7%) had knowledge of Pap smear test. Two hundred and sixty (35.3%) of the respondents know that early detection of HPV can prevent cervical cancer and in spite of this, only 110 (14.9%) have taken the Pap smear test before while 151 (20.5%) are not willing to go for the test at all.* Conclusions*. There is therefore the need to create proper awareness on the HPV and its possible consequence of cervical carcinoma.

## 1. Introduction

HPV infection, a sexually transmitted infection, occurs throughout the world and has been implicated as the principal cause of cervical cancer. More than 100 HPV types have been identified and most types infect the cutaneous epithelium where they cause common skin warts. About 40 types infect the mucosal epithelium; these are categorized according to their epidemiologic association with cervical cancer [1].

Worldwide, cervical cancer is one of the most common cancers among women. It is estimated that almost half a million new cases and 274,883 deaths occurred in 2008, and about 86% of these cases occur in developing countries [2]. Worldwide, cervical cancer mortality rates are lower than incidence with a ratio of mortality to incidence of 52%. The crude incidence rate of cervical cancer is 19.3 in Nigeria, 19.9 in Western Africa, and 15.8 worldwide. In Nigeria, it has recently been estimated that, every year, 14,550 new cases of cervical cancer are diagnosed and 9,659 die from the disease. Cervical cancer occurs rarely in women under 30 years of age and occurs most commonly in women over 40. Since it is also associated with the early age at which sexual intercourse begins, it remains a problem in African countries.

Few years ago, some new vaccines were discovered against the HPV infection and cervical cancer which has transformed the prospects of reducing HPV incidence on a global scale [3]. The vaccines contain a combination of genetic material from more than one origin. Since the vaccines do not contain any live biological product or DNA, they are noninfectious and the HPVs targeted by the vaccines are “high risk” types 16 and 18 and “low risk” types 6 and 11 [4]. HPV vaccines have also been shown to prevent precursors to some other cancers associated with HPV [5]. HPV vaccines induce high levels of serum antibodies in virtually all vaccinated individuals and are generally well tolerated with minor adverse events at the injection site like pain, erythema, and oedema. [6]. HPV vaccines are targeted at girls and women of ages 9 to 26 because it is only useful if given before infection occurs. This is because the HPV vaccines are designed to be prophylactic (i.e., to prevent infection and consequent disease), not therapeutic. Therefore, public health workers target girls before they begin having sex especially in deprived areas [7].

The vaccine against HPV types 6, 11, 16, and 18 has been licensed but is currently not available in Nigeria due to importation, distribution, and other regulatory requirements, as well as price negotiations issues. However, a vaccine which targets both HPV strains 16 and 18 and has proven to be 92% effective with more than four-year potency is both licensed and available in Nigeria [6]. In April 2009, WHO issued a position paper on HPV vaccination which recommended that routine HPV vaccination be included in national immunization program, provided that cervical cancer or other HPV-related disease prevention measures are a public health priority for the country [7]. The vaccine is available and accessible at some private and public hospitals in Nigeria at a cost range of nine thousand naira to fifteen thousand naira (N9, 000:00–N15, 000:00) [8].

Despite the prevalence and burden of cervical cancer worldwide with almost 80% occurring in developing countries such as Nigeria, only about 52% of Nigerian women were aware of this deadly disease [9]. Less than 7.1% of Nigerian women have reportedly had cervical cancer screening done and only 8% of women who attended the clinic between 2010 and 2011 had HPV vaccination.

The effectiveness of a vaccine delivery program depends largely upon the awareness of the vaccine and the attitude in terms of acceptability of the vaccine [6]. This study was therefore carried out to assess the awareness on HPV and acceptability of the cervical cancer vaccine and that of the Pap smear test among the general population of women in Nigeria.

## 2. Methodology

This was a questionnaire-based cross-sectional survey carried out in Oyo and Osun States both in southwestern Nigeria. The study population included randomly selected women aged 18 years and above as at the time of the administration of questionnaire. Face-to-face structured interviews were conducted after the purpose of the study had been explained to the participants and their informed consent sought and gained. Women who did not give their consent were excluded from this study. The survey was conducted from September 2013 to March 2014. A standardized questionnaire assessing knowledge of HPV, cervical cancer, Pap smear test, vaccine acceptability, and willingness to participate in HPV vaccination as well as demographic characteristics related to HPV and cervical cancer was administered to a total of 737 women.

## 3. Results

### 3.1. Demographic Characteristics

Seven hundred and thirty-seven questionnaires were administered in face-to-face interviews. The mean age of respondents was 29.3 years (range: 18–54). Three hundred and forty-seven (47%) were single while 390 (52%) were married. Two hundred and fifty-one (34%) of the respondents were students, 181 (24%) civil servants, 129 (17%) into a trade or private business, 37 (5%) artisans, 57 (8%) housewives, and 82 (11%) others that may include the unemployed (Table 1).

Sixty-one (8.3%) had primary education, 203 (27.5%) had secondary education, and 447 (60.7%) had tertiary education while 26 (3.5%) had no education at all. Three hundred and sixty-five (49.5%) were Christians while the rest 372 (50.5%) were Muslims. Six hundred and eighteen (83.9%) of the participants were of the Yoruba tribe, 65 (8.8%) Igbo, 42 (5.7%) Hausa, and the remaining 12 (1.6%) others that may include tribes such as Edo, Tiv among others.

This study shows that sociodemographic characteristics of individuals such as age (*P* = 0.039), marital status (*P* = 0.0), occupation (*P* = 0.0), level of education (*P* = 0.002), and religion (*P* = 0.0) have significant effects on their willingness to partake in research studies.

### 3.2. Attitude and Practice

As per number of sexual partners, 64 (8.7%) of the respondents have no sexual partners, 421 (57.1%) had between 1-2 partners, and 103 (14.0%) have more than 3, while the rest 149 (20.2%) did not disclose.

### 3.3. Knowledge about HPV, HPV Vaccine, and Pap Smear

One hundred and seventy-six (23.9%) of the respondents had knowledge of HPV, 474 (64.3%) are aware of cervical cancer, 200 (27.1%) are aware of the HPV vaccine, 300 (40.7%) had knowledge of Pap smear test, 136 (18.5%) are aware that HPV causes cervical cancer, and 260 (35.3%) know that early detection prevents cervical cancer and in spite of this, only 110 (14.9%) have taken the Pap smear test before (Table 2).

The sources of information in the respondents include health care workers 178 (24.2%), television and radio 320 (43.4%), Internet 86 (11.7%), newspaper 28 (3.8%), and others 53 (7.2%) which may include school, friends.

Four hundred and seventy (63.8%) of the respondents were willing to take the test in future, of which the rest 116 (15.7%) were not at all willing to take the test and 151 (20.5%) were not sure. Of the respondents, 569 (77.2%) were willing to take the HPV vaccine and 76 (10.3%) were unwilling, while the rest 92 (12.5%) are not sure if they would take the vaccine; this is because most are not sure the vaccine will be able to protect them against cervical cancer.

## 4. Discussion

This study revealed that the level of awareness of the HPV vaccine is low (27.1%) among the general population of women in southwest Nigeria; however after they were enlightened, 77.2% were willing to take the vaccine. This contradicts 62.7% awareness level reported in another study [10]. The knowledge of cervical cancer (64.3%) seems to be higher than the knowledge about HPV (23.9%) and also most respondents (81.5%) are not aware that HPV causes cervical cancer. A similar study carried out in Ibadan reported a 67.6% level of awareness of cervical cancer and contrary to our reports 83.3% level of awareness of HPV [11]; also in Owerri, 85.9% level of awareness of cervical cancer was reported [12].

Only 14.9% respondents have taken the Pap smear test before; this shows that the prevalence rates of cervical cancer in Nigeria may be underreported, since most women in this study do not know their status, even though a good majority of them (>90%) have at least a primary education. Most of the knowledge in respondents came from television and radio (43.4%) and the least from newspapers (3.8%); this study also shows that information delivery through the health care system is poor (24.2%). This report contradicts that of another study which reports that knowledge of HPV and cervical cancer in respondents came from the health care systems [11]. However there have been several reports on other diseases, where the media (television, radio, and the Internet) have been identified as a major source of health related information [10].

From this study, it is evident that sociodemographic characteristics of individuals such as age, marital status, occupation, level of education, and religion have significant effects on their willingness to partake in research studies such as this, and this has implication on the relevance of results from similar research studies.

## 5. Conclusion

Knowing the fact that cervical cancer if unattended may lead to more serious complications or even death, there is thus an urgent need for educating Nigerian women of child-bearing age and postmenopausal women who are not likely to get regular Pap smear tests, as well as a need to advocate for an increase in the availability and affordability of immunization facilities across the country.

The level of awareness is low, so prior to the introduction of HPV vaccine in the national immunization scheme, there should be more awareness created; this should involve not only health workers but the media as well as other stakeholders at the grassroots. This would increase the acceptability of the vaccine and hence the success of immunization program (Table 3).

Information is power, so there is need to reorientate Nigerian women on the need to take the Pap smear test in order to know their status as this is very important for a successful nationwide immunization program. If more women take the Pap smear test and know their status, then we might have a true estimate of the prevalence levels of cervical cancer and this will help stakeholders in decision making. It is necessary however to reemphasize the efficacy of the available HPV vaccines as this would increase the willingness to take the vaccines.

## Figures and Tables

**Table 1:** Demographic characteristics.

Characteristic	Oyo (*N* = 285) No	%	Osun (*N* = 452) No	%	*χ* ^2^	*P* value
Age						
18–25	102	35.8	200	44.2	10.09	0.039
26–35	123	43.2	153	33.8
36–45	40	14.0	76	16.8
46–55	15	5.3	14	3.1
>55	5	1.7	9	2.0
Marital status						
Single	110	38.6	237	52.4	13.43	0.000
Married	175	61.4	215	47.6		
Occupation						
Student	51	17.9	200	44.2		
Civil servant	95	33.3	86	19.0	60.93	0.000
Business/trade	56	19.6	73	16.2
Artisan	15	5.3	22	4.9
Housewife	33	11.6	24	5.3
Others	35	12.3	47	10.4
Educational qualification						
Primary	36	12.6	25	5.5		
Secondary	86	30.2	117	25.9	15.35	0.002
Tertiary	154	54.0	293	64.8
None	9	3.2	17	3.8
Religion						
Christianity	167	58.6	198	43.8	15.3	0.000
Islam	118	41.4	254	56.2		
Ethnicity						
Yoruba	248	87.0	370	81.8		
Igbo	20	7.0	45	10.0	3.72	0.294
Hausa	14	4.9	28	6.2
Others	3	1.1	9	2.0
Number of sexual partners						
None	17	6.0	47	10.4		
1-2	185	65.0	236	52.2	52.52	0.000
>3	58	20.2	45	10.0
Didn't say	25	8.8	124	27.4

*P* value <0.05 is considered significant.

**Table 2:** Knowledge of HPV, HPV vaccine, and Pap smear test.

	Oyo (*T* = 285)		Osun (*T* = 452)		*χ* ^2^	*P* value
	Yes (%)	No (%)	Yes (%)	No (%)		
HPV^a^	62 (21.8)	223 (78.2)	114 (25.2)	338 (74.8)	1.16	0.282
Cervical cancer	198 (69.5)	87 (30.5)	276 (61.1)	176 (38.9)	5.39	0.02
HPV vaccine	69 (24.2)	216 (75.8)	131 (29.0)	321 (71.0)	2.01	0.156
CC^b^ screening	166 (58.2)	119 (41.8)	134 (29.6)	318 (70.4)	59.23	0.000
Cause of CC	38 (13.3)	247 (86.7)	98 (21.7)	354 (78.3)	8.09	0.004

Early detection prevents CC	132 (46.3)	153 (53.7)	128 (28.3)	324 (71.7)	24.8	0.000

^a^Human papilloma virus.

^b^Cervical cancer.

**Table 3:** Acceptability of Pap smear test and HPV vaccine.

	Oyo (*T* = 285)			Osun (*T* = 452)			*χ* ^2^	*P* value
	Yes (%)	No (%)	Not sure (%)	Yes (%)	No (%)	Not sure (%)		
CC^a^ screening	205 (72.0)	32 (11.2)	48 (16.8)	265 (58.6)	84 (18.6)	103 (22.8)	9.93	0.002
HPV^b^ vaccine	251 (88.1)	20 (7.0)	14 (4.9)	318 (70.4)	56 (12.4)	78 (17.2)	8.72	0.003

Previous testing	65 (22.8)	220 (77.2)	—	45 (10.0)	407 (90.0)	—	22.73	0.000

^a^Cervical cancer.

^b^Human papilloma virus.
